# Preference for high-carbohydrate foods does not change for children and adolescents in insulin-induced hypoglycemia

**DOI:** 10.1136/bmjdrc-2022-003065

**Published:** 2022-11-08

**Authors:** Sarah Sauchelli, Peter J Rogers, George Fry, Julian P Hamilton-Shield

**Affiliations:** 1 NIHR Bristol Biomedical Research Centre, University Hospitals of Bristol and Weston NHS Foundation Trust and University of Bristol, Bristol, UK; 2 School of Psychological Sciences, University of Bristol, Bristol, UK; 3 Patient co-author, Bristol, UK

**Keywords:** Food Choice, Diabetes Complications, Hypoglycemia, Carbohydrates

## Abstract

**Introduction:**

Hypoglycemia elicits coordinated counter-regulatory neuroendocrine responses. The extent to which this process involves an increased drive to eat, together with greater preference for foods high in carbohydrate content, is unclear. Our objective was to examine this effect in children and adolescents (age 5–19 years) without diabetes and no prior known experience of hypoglycemic episodes.

**Research design and methods:**

We administered a computerised task designed to examine preference for high-carbohydrate foods (sweet and savory) to pediatric patients (n=26) undergoing an insulin tolerance test as part of the routine clinical assessment of pituitary hormone secretory capacity. The task was completed at baseline and three time points after intravenous infusion of insulin (approximately 7, 20 and 90 min).

**Results:**

Although all patients reached insulin-induced hypoglycemia (mean venous glucose at nadir=1.9 mmol/L), there was moderate evidence of no effect on preference for high-carbohydrate foods (moderate evidence for the null hypothesis) compared with euglycemia. Patients also did not display an increase in selection of foods of high compared with low energy density. Sensitivity of the task was demonstrated by decreased preference for sweet, high-carbohydrate foods after consumption of sweet food and drink.

**Conclusions:**

Results support the view that acute hypoglycemia does not automatically prompt the choice of high-carbohydrate foods for rapid glucose restoration, and further stresses the importance that people and families with children vulnerable to hypoglycemic episodes ensure that ‘rapidly absorbed glucose rescue therapy’ is always available.

**WHAT IS ALREADY KNOWN ON THIS TOPIC**

- People with insulin-dependent diabetes report having difficulties in self-treating a hypoglycemic episode, despite receiving diabetes education.
- It is unclear whether the healthy neuroendocrine counter-regulatory response to hypoglycemia modulates preference for foods that can rapidly restore glucose availability.

**WHAT THIS STUDY ADDS**

- This is the first study to evaluate the effect of insulin-induced hypoglycemia on the food choice of children and adolescents without diabetes or prior known experience of hypoglycemic episodes.
- The study demonstrates that glucose depletion does not independently direct decision-making towards foods with a fast glucose absorption rate.

**HOW THIS STUDY MIGHT AFFECT RESEARCH, PRACTICE OR POLICY**

- Adequate diabetes education and preparation for possible hypoglycemic episodes continue to be the best approach to accurate treatment of hypoglycemia.

## Introduction

Despite advances in technologies designed to facilitate the monitoring and management of blood glucose in patients with diabetes mellitus, avoidance of hypoglycemia continues to be a challenge for patients with type 1, and those with insulin-dependent type 2 diabetes.^1^ Despite clear guidance on recommended treatment,^2^ people with diabetes can struggle to self-treat a hypoglycemic episode correctly; some develop hyperglycemia due to overeating, and others experience severe, prolonged hypoglycemia by selecting foods that slow absorption of glucose (eg, peanut butter).^3–5^ Consequences of inappropriately and inadequately treated hypoglycemia can include seizures, coma, and death.

Under normal circumstances, hypoglycemia is expected to elicit a counter-regulatory hormonal response comprising curtailed pancreatic B-cell insulin secretion, a rise in pancreatic B-cell glucagon and adrenomedullary epinephrine secretion, together with an increased drive to eat.^6 7^ Hypoglycemic Sprague-Dawley rats have been found to eat more food than their counterparts given a saline as opposed to a glucose solution.^8^ Earlier research in adults with type 1 diabetes suggested that insulin-induced hypoglycemia elevates desire for foods high in carbohydrate more than it does for desire for low-carbohydrate foods.^9^

A tendency to seek carbohydrate-loaded food in response to hypoglycemia appears to align with models of regulation of food preferences rooted in glucose metabolism.^10 11^ These posit that glucose availability plays a fundamental role in the experience of hunger, meal initiation, and satiety. A fall in blood sugar detected by glucose-sensing brain regions^12^ is argued to prompt the individual to seek foods with a high glycemic load, whereas insulin secretion through glucose absorption to signal satiety and meal termination via the release of glucagon-like peptide-1.^11^ Hence, it is reasonable to expect that a considerable reduction in the availability of glucose in the bloodstream would increase preference for high-carbohydrate foods until euglycemia is achieved.

However, if glycemia does exert tight control on short-term eating behavior, we would expect people with diabetes to be able to treat their hypoglycemic episodes effectively. The challenge may be partially attributed to the individual’s hypoglycemia awareness.^7 13^ In the study by Strachan and colleagues,^9^ the 13 participants living with type 1 diabetes had normal hypoglycemia awareness (experience or perceive symptoms of hypoglycemia) and no history of severe hypoglycemia. Poor glucose control or long-standing recurrent hypoglycemia can impair the ability to detect the onset of hypoglycemia,^7 14^ and alter the behavioral response. However, previous work has shown that rats with an impaired neuroendocrine counter-regulatory response can also present a hyperphagic response to insulin-induced hypoglycemia, leading the authors to conclude that eating response to hypoglycemia may be regulated by separate neural substrates and pathways to the neuroendocrine response.^8^

Diabetes education reduces the risk of severe hypoglycemia.^15 16^ Cognitive deterioration due to the rapid decline in brain glucose availability^6^ may interfere with/disrupt an individual’s ability to follow advice and self-treat hypoglycemia. Though the effects of glucose on cognitive function are not entirely clear,^17^ people with diabetes have attributed difficulties in self-treating to ‘confusion’, ‘disorientation’, and ‘panic’.^4^ Individuals report being unable to restrain their eating to the amounts needed, and to overindulge in foods they like.^4^ Another study shows hypoglycemia-related increases in food intake in healthy adults without diabetes are due to an increased consumption of foods high in fat content and low glycemic index.^18^ These findings are inconsistent with the notion of increased preference for carbohydrates triggered automatically by hypoglycemia, and are aligned with critique of the glucostatic theory of appetite control.^19^ Instead, nutritional knowledge, individual food preferences, and learned eating habits may also be at play in an individual’s behavioral response to hypoglycemia.

Uniquely, the present study revisited the impact of glucose homeostasis on eating predispositions in children and adolescents (age 5–19 years) without diabetes or prior experience of hypoglycemic episodes. To overcome pragmatic and ethical constraints of testing in free living conditions, we administered a computerised forced-choice food preference task used in nutrition research^20^ to children and adolescents undertaking an insulin tolerance test (ITT) as part of a clinical assessment of pituitary hormone secretory capacity. We hypothesized that participants’ choice of high-carbohydrate foods over low-carbohydrate foods (matched for calories) would increase when in a hypoglycemic state. We additionally hypothesized that the number of gummy bears participants would eat (one of the recommended items to treat hypoglycemia) would be increased during hypoglycemia. Finally, as food preferences and eating habits are shaped by development,^20 21^ we analyzed whether age moderates the effect of hypoglycemia on eating predispositions.

## Research design and methods

### Participants

This repeated-measures, single-cohort, experimental study was carried out at the Clinical Investigation Unit of the Bristol Royal Hospital for Children. The hospital is one of the few in the UK that carries out ITTs in children, the gold-standard procedure for assessing growth hormone secretion.

Participants were pediatric outpatients at the Bristol Royal Hospital for Children undertaking an ITT to evaluate growth hormone function (either to inform diagnosis of growth hormone deficiency or assess discontinuation of growth hormone replacement therapy). Participants did not have diabetes. Recruitment took place from February 2019 until April 2021, with a pause between March and July 2020 due to the COVID-19 pandemic. Based on the effect sizes of previous research in adults,^9^ we estimated (using G*Power V.3.1.9.4) that 36 participants would be needed to detect a similar effect size (mean d=0.976; 21) with a two-tailed test, an α of 0.05, and 80% power.^22^

Patients were recruited in the study if they were between 5 and 19 years of age and were receiving an ITT as part of a growth hormone assessment for poor linear growth at the Bristol Royal Hospital Information. Exclusion criteria were as follows: (a) a history of cancer therapy, as it may alter the patient’s counter-regulatory endocrine responses and nervous autonomic control^23^; (b) presence of ischemic heart disease, epilepsy, unexplained blackouts and cardiac arrhythmias, as these medical conditions are strong contraindications for the ITT; (c) presence of neurological impairments/symptoms that may interfere with the patient’s ability to complete assessment; (d) a diagnosis of diabetes mellitus or a history of recurrent hypoglycemic episodes.

### ITT procedure

**Figure 1:**
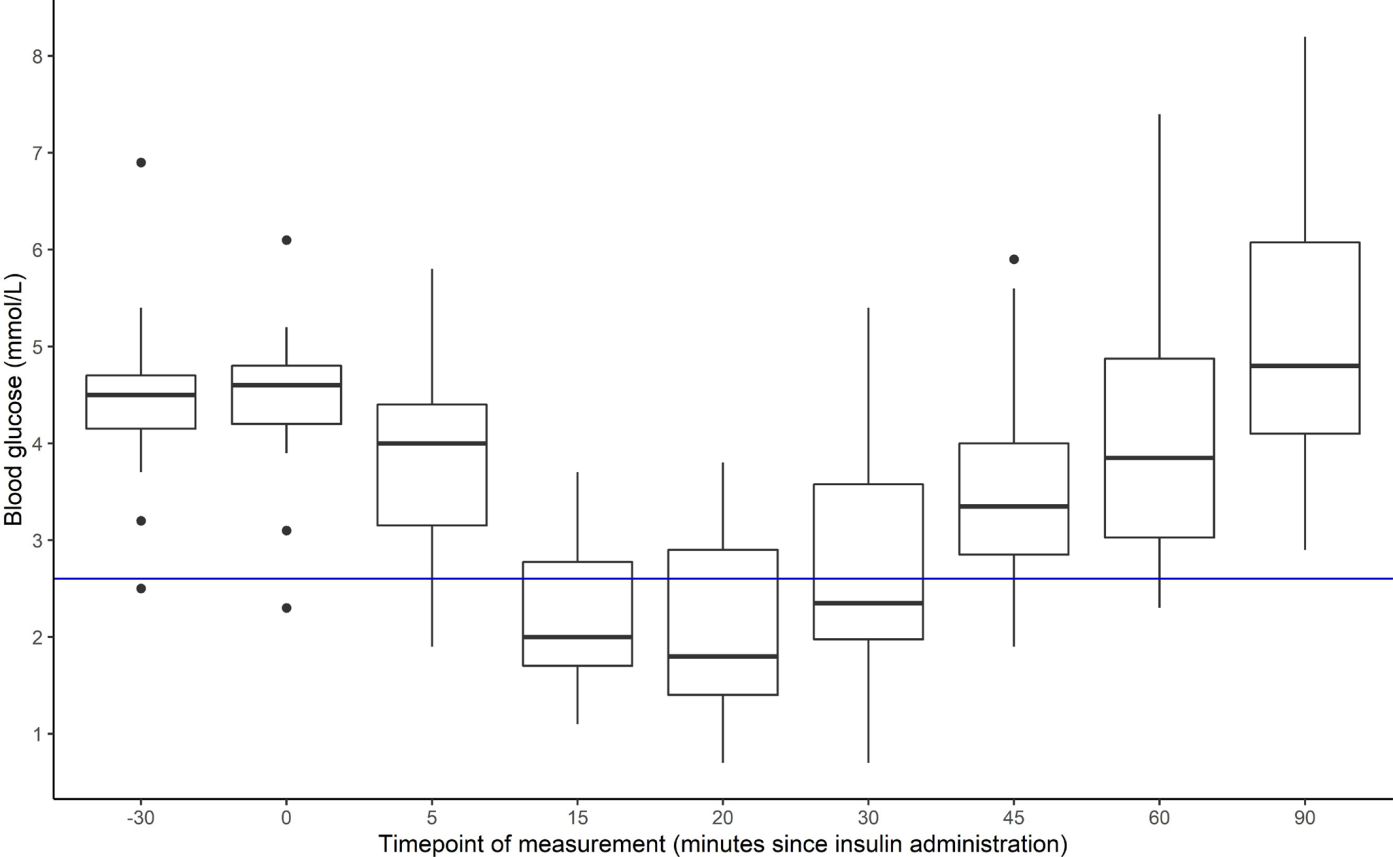
Blood glucose response to administration of insulin. Blue line: clinical threshold for hypoglycemia (2.6 mmol/L).

Figure 1 depicts the protocol of the ITT. Patients arrive at the Clinical Investigation Unit between 08:00 and 09:00, after an overnight fast. The patient remains supine throughout the test. Height and weight are measured, and pulse oximetry is completed. Following consent to the ITT (provided by the parent/guardian if under the age of 16 years, the patient if aged 16 years or above), a cannula is inserted in the non-dominant arm or another suitable location in the upper limbs for repeated blood sampling. Thirty minutes after cannulation, a dose of Actrapid insulin corresponding to the patient’s body weight (0.15 units/kg) is administered intravenously. Blood samples are collected at time −30 min to insulin administration, 0, +15, +30, +45, +60, +90, and +120, to measure glucose, cortisol and growth hormone concentrations. Additional samples may be collected as needed to monitor glucose, using a One Touch Glucose Meter. The cannula is flushed with 5 mL 0.9% sodium chloride before and after each sampling and insulin administration.

When glucose readings reach the medically defined threshold for hypoglycemia (<2.6 mmol/L), patients are treated with a squash drink (500 mL: 50% water, 50% fruit concentrate) and five digestive biscuits. When necessary, one or more doses of glucose (2 mL/kg of intravenous 10% glucose) may be administered to accelerate recovery. The test is terminated if the patient’s consciousness levels fall. An observation sheet is used to record symptoms of hypoglycemia and treatment. Blood samples are sent to the laboratory for analysis.

### Food preference task

A computerised two-alternative forced-choice task was administered on four occasions to measure food preferences across the ITT. Eighteen foods were selected to permit analysis of possible patterns in food preferences governed by the foods’ properties: energy density, carbohydrate content (high or low), and taste (sweet/savory). Suitability of the selected foods for the target group was subsequently checked against reports of common foods eaten by children in the UK and consultation with the Bristol Young Persons Advisory Group. Thirty-two images of food were presented to the group, who individually rated how much they liked the foods in a 10-point Visual Analogue Scale. Additional questions were asked regarding the inclusion of labels, display clarity, and presentation style. All food images were presented on the same white plate, photographed at a 45° angle, without any packaging, with the same plain background (wooden surface, white panel), the same lighting and no filters. The least liked food images by the majority of the group were removed, to achieve a set of 18 food images, with portion size being determined by a balance between gradual increments in energy composition and normal portions eaten. Further details of the foods are provided in online supplemental material 1. One participant reported being vegetarian and proceeded to complete an adapted version of the task displaying vegetarian alternatives to the meat products. Participants who only ate halal meat (n=2) opted for assuming that all meat was halal rather than completing the vegetarian version of the task. In the computerised task, participants were presented (side-by-side) with a combination of every food pair possible (total trials=153), were asked ‘Imagine you can have one of these foods to eat right now. Which one would you choose? Only these portions are available’, and had to select the food on the left or right using the corresponding arrow keys on the keyboard. Presentation of food pairs was randomized.

10.1136/bmjdrc-2022-003065.supp1 Supplementary data

At the end of this task, participants were presented with images of a plate containing gummy bears, a food often recommended to people living with type 1 diabetes to quickly raise blood sugar levels when experiencing a hypoglycemic episode. Presentation was alike that of the other food images, except the photographs had been taken at a 90° angle. Using the right and left arrow keys, participants could increase or decrease the number of gummy bears on the plate. They were instructed to select ‘the amount of gummy bears you would eat right now’. This task was set to evaluate the quantity of fast-acting carbohydrates people would naturally eat in response to hypoglycemia, without prior knowledge on recommended amounts.

### Study protocol

The study procedure was timed and adapted on a patient-by-patient basis to align with the ITT. Participants were recruited either by responding to a letter invitation sent by the clinical team alongside the ITT appointment letter or by talking directly with the researcher (SS) on the day of the ITT after an introduction from the nurse/registrar. Informed consent was obtained once the clinical team confirmed that the ITT was going ahead. Patients aged 16 years and above were given an age-appropriate information sheet and provided written informed consent after having an opportunity to ask further questions. Patients aged less than 16 years received an age-appropriate information sheet, and the information was also verbally explained to each patient in a conversation format to ensure that the patient had understood. The parent/guardian also received a more comprehensive participant information sheet. After the opportunity to ask questions, the patients were supported in filling in an age-appropriate assent form if they were happy to take part, and the parent/guardian signed the consent form on their behalf. The age thresholds reflect the clinical consent procedures adopted by the Bristol Royal Hospital for Children.

Participants were asked to verbally indicate how hungry they felt on a scale from 0 (‘not hungry at all’) to 10 (‘extremely hungry’), and the food item they would most like to eat at that point in time. Presence of allergies or foods that the child did not eat were noted down. The food preference task was then administered at the following time points: (a) baseline: around −20 min before insulin administration; (b) possible hypoglycemia: +7 min (with expected completion at around +14); (c) possible hypoglycemia: +20 min (with expected completion at around +25); (d) euglycemia: +90 min, before lunch. Given that hypoglycemia can result in impaired cognitive function,^6^ participants completed a 1-minute visual search task before the second and third administration of the food preference task. After the final administration, participants were once again asked about their hunger levels and food they would like to eat the most, and their feedback on the task. With the help of the parent/guardian, participants indicated how familiar they were with each of the foods in the computerised task. Weight, height, and the amount of squash and biscuits consumed during the procedure were noted down. Withdrawal of the participant from the study took place if the ITT had to be terminated early, the participant no longer wanted to complete the task (to be expected given the symptoms generated by hypoglycemia) or the study had to be stopped to ensure successful completion of the clinical procedure.

### Statistics

Analyses were primarily conducted in R.^24^ When null findings were obtained, SPSS (V.28) was used to run Bayesian-related samples inference. Matching of venous blood glucose with output from the computerised food preference task was performed posterior to the ITT once glucose levels had been reported by the hospital laboratory. The first and fourth rounds of administering the computerised task were matched with the blood glucose readings taken 30 min before insulin infusion and 90 min after. We selected either the second or third round to evaluate food preferences in hypoglycemia using the following criteria: (a) lowest venous glucose level (ideally below 2.6 mmol/L), (b) prior to ingestion of biscuits and squash (standard treatment), (c) prior to intravenous glucose infusion (occasional). The corresponding venous glucose levels were extracted for comparison across the three time points of interest: fasted euglycemia (baseline), hypoglycemia, and treated euglycemia (recovered).

Repeated measures analysis of variance (ANOVA) was used to compare frequency with which participants selected high-carbohydrate sweet and high-carbohydrate savory foods over alternatives in the food preference task. Partial η^2^ was used for estimates of effect size, interpreted using the thresholds provided by Miles and Shevlin^25^ (small=0.01, moderate=0.06, large=0.14). Post-hoc pairwise comparisons were carried out using Bonferroni adjusted t-tests and the 95% CIs were calculated. Age was entered as a covariate. Due to violation of ANOVA assumptions, Friedman tests were performed to analyze differences across time points in blood glucose and number of gummy bears selected. Effect size estimates were calculated using Kendall’s W, which relies on Cohen’s interpretation guidelines (0.1–<0.3=small, 0.3–<0.5=moderate, ≥0.5=large^26^). Bonferroni-adjusted pairwise comparisons were carried out with Wilcoxon signed-rank test where appropriate and 95% CIs calculated. In addition, Pearson’s correlations were conducted to assess the relationship between arterial glucose at hypoglycemia and selection of high-carbohydrate foods. Descriptive data were summarized using means and SDs. To maintain consistency, means and SDs were also calculated in cases where non-parametric tests were carried out.

## Results

Of 36 patients recruited into the study, 8 did not complete assessment due to: a change in the date of the ITT (n=3), participant withdrawal during the study (n=2), inability to engage with the computerised food preferences task (n=1), presence of severe symptoms of hypoglycemia requiring immediate clinical attention (n=2). Further, analysis was not possible for one participant due to an error in the computerised task, and for one participant because only interstitial glucose data could be extracted. Data from a total sample of 26 participants were included for analyses. Participant characteristics are presented in table 1.

**Table 1:** Demographic characteristics of sample included for analysis

	N=26*
Male	16 (62%)
Age (years)	15 (10–16)
Height (cm)	149 (122–166)
Weight (kg)	36 (25–59)
Weight status (BMI percentile for age, ref)	
Underweight	4 (15%)
Healthy weight	18 (69%)
Overweight	1 (3.8%)
Obesity	3 (12%)
Growth hormone response	
Deficiency	15 (58%)
Sufficiency	11 (42%)
Cortisol response	
Deficiency	9 (35%)
Sufficiency	17 (65%)

*n (%); median (IQR).

BMI, body mass index.

### Response to insulin administration

Figure 1 shows venous glucose across the ITT. Glucose concentrations were generally in the normal range in the time preceding intravenous insulin administration, but there was a drop to hypoglycemia in the 15–30 min after. By the end of the ITT procedure, which included treatment with food and drink and for 20 participants intravenous glucose administration (twice for two participants), all participants had returned to euglycemia. Mean time to hypoglycemia was approximately 17 min (±6.1 min), except for one participant who was already in hypoglycemia at the time of insulin administration (not detected at testing due to disparities between interstitial and venous glucose results). The main reported symptoms during ITT were tiredness (n=6), feeling faint/paleness (n=5), and dizziness (n=4). Only one participant reported elevated hunger not attributable to time since last having eaten (all children had been fasted overnight).

Analysis of glucose responses was further split by participants’ ITT results, but trends were similar regardless of the presence of growth hormone or cortisol deficiency or not (see online supplemental material 2).

10.1136/bmjdrc-2022-003065.supp2 Supplementary data

On average, participants consumed 93% (±18.4%) of given squash, and 4.3 (±1.0) biscuits to treat hypoglycemia. Twenty-one participants were additionally treated with intravenous glucose administration (2 mL/kg of 10% dextrose), of whom one received two doses.

### Food preferences

Data used to evaluate food preferences during hypoglycemia were obtained from the second round of completing the computerised task for 20 participants (77%) and the third round for six participants (23%). The first and last rounds were used for comparison to euglycemia (baseline, before insulin administration after an overnight fast; and follow-up, 90 min after insulin administration and treatment).

Venous glucose was found to differ across the three time points of interest (X^2^(2)=40.1, p<0.001; W=0.8). Bonferroni-adjusted pairwise Wilcoxon signed-rank test between time points revealed that concentrations were similar at baseline (4.5 mmol/L; SD=0.8 mmol/L) and follow-up (5.1 mmol/L; SD=1.4 mmol/L; Z(26)=89, p=0.08, 95% CI: −1.35 to 0.05), and both were elevated compared with the critical assessment mid-ITT, when participants were in hypoglycemia (1.9 mmol/L, SD=0.5 mmol/L; vs baseline: Z(26)=351, p<0.001, 95% CI: 2.25 to 2.80; Z(26)=351, p<0.001; 95% CI: 2.55 to 3.80).

**Figure 2:**
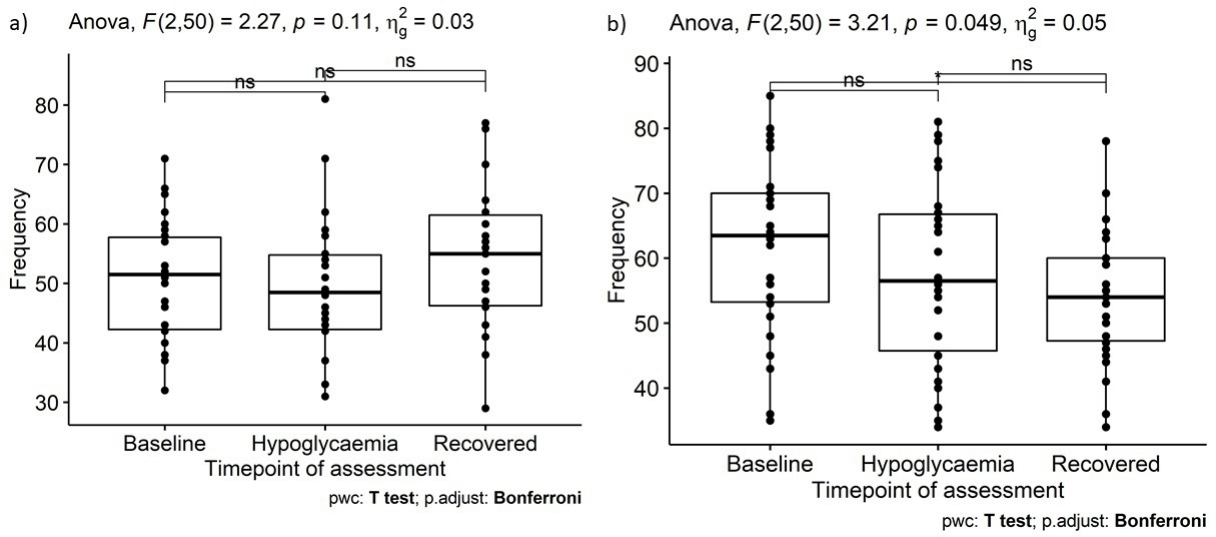
Number of times participants (n=26) selected images of foods that were (A) high in carbohydrate and savory, and (B) high carbohydrate and sweet in the food preference task. Data are split across assessment time point: before insulin administration (baseline), when experiencing hypoglycemia (venous glucose <2.6 mmol/L, except for one participant with concentrations of 2.7 mmol/L), and at the end of the ITT after appropriate treatment (recovered). ANOVA, analysis of variance; ITT, insulin tolerance test.

The number of savory high-carbohydrate foods selected did not differ between the three time points (F(2, 50)=1.60, p=0.21, η^2^=0.02, figure 2A). A Bayes factor of 5.52 was obtained for mean difference between baseline and hypoglycemia (mean difference: 1.2, t(25)=−0.76, p=0.46; 95% CI: −2.2 to 5.2), indicating moderate evidence of null effects. The number of sweet high-carbohydrate foods selected also did not change between baseline and hypoglycemia (mean difference: −7.3, t(26)=0.76, p=0.45, 95% CI: −9.33 to 5.02; figure 2B), with a Bayes factor of 5.02 for the difference, indicating moderate evidence of null effects. By contrast, the number of sweet high-carbohydrate foods selected decreased between baseline and recovery from hypoglycemia (mean difference −7.3, 95% CI: −12.9 to −1.6, t(25)=2.65, p=0.014, Cohen’s d=−0.52; figure 2B).

Furthermore, Pearson correlations demonstrated that venous glucose concentrations reached by participants at hypoglycemia did not correlate with an increased selection of high-carbohydrate foods (savory: r(24)=−0.04, p=0.84; sweet: r(24)=−0.02, p=0.94). A repeated-measures ANOVA showed that age did not impact how often participants selected high-carbohydrate, savory food images in the computerised task (F(1, 48)=0.19, p=0.83, η^2^<0.01), nor selection of high-carbohydrate, sweet foods (F(1, 48)=1.02, p=0.37, η^2^=0.02) across time points.

Additional analyses were carried out to test the exploratory hypothesis that hypoglycemia would increase selection of energy-dense (rich) foods. When foods were grouped into high and low energy density, the number of the high-energy dense foods selected did not differ across time points (F(2, 50)=0.70, p=0.50, η^2^=0.01). A Bayes factor of 5.20 was obtained for mean difference between baseline and hypoglycemia (mean difference: −1.1, t(25)=−0.70, p=0.49; 95% CI: −4.41 to 2.26), indicating moderate evidence of a null effect. However, a curvilinear trend was found with selection of individual food choices on the basis of energy density (see online supplemental material 3).

10.1136/bmjdrc-2022-003065.supp3 Supplementary data

### Food quantity

**Figure 3:**
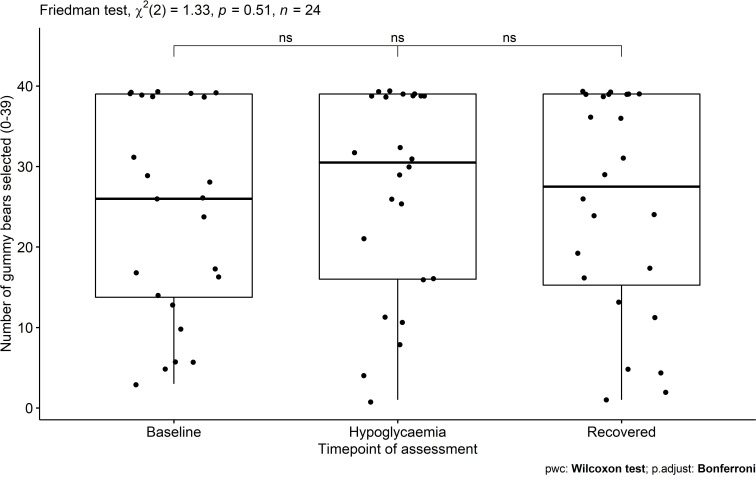
Number of gummy bears participants (n=24) selected at each time point of assessment. ns, not significant.

Data for selection of gummy bears had to be removed for two participants due to technical issues. Eight participants selected the maximum number of gummy bears at baseline, 10 during hypoglycemia, and 7 after recovery. A Friedman test revealed mean number of gummy bears selected to be similar across time points (X^2^(2)=1.3, p=0.51, W=0.03; figure 3).

## Conclusions

Understanding how food choices are made during hypoglycemia is fundamental to improve treatment in people with diabetes requiring insulin. We administered insulin intravenously to children and adolescents, and found moderate evidence that acute hypoglycemia does not result in a preferential shift towards high-carbohydrate foods. Our findings also suggest that decision-making on quantity of high-carbohydrate food to ingest is likely to be unaffected by venous glucose availability. These results contradict ‘glucostatic’ theories of short-term regulation of food preferences and elevate the importance of patient preparation for hypoglycemia.

Data challenging the glucostatic theory of appetite regulation have been reported previously.^18 27^ We have also found moderate evidence of a null effect, thus findings should be interpreted with some caution. The task however did reveal a reduction in preference for sweet but not savory high-carbohydrate foods after hypoglycemia was treated with squash and biscuits. This shift in taste preference is consistent with the well-established phenomenon of sensory-specific satiety^28^ that describes a reduction in preference for recently eaten versus uneaten foods based on their taste qualities.^29 30^ The ability of our task to expose sensory-specific satiety suggests that it is sensitive to temporal shifts in food preferences, and further data collection using this task could strengthen the evidence of the null effect. The task also revealed a curvilinear relationship between food selection and food energy density, which confirms observations using other measures and foods in adult participants.^31^ This further confirms the sensitivity of our current methods and provides additional evidence for food choice being determined by a complex interplay of multiple factors, rather than glucose availability alone.

It is noteworthy that mild hypoglycemia activates limbic-striatal brain regions associated with motivation and increased desire for high-calorie foods,^32^ and in patients with type 1 diabetes it is related to changes in the medial orbitofrontal cortex only in response to high-calorie foods.^33^ An alternative hypothesis therefore is that hypoglycemia triggers an attentional shift towards high-calorie foods rather than foods with a higher ratio of carbohydrate content. We found moderate evidence against this effect. Nonetheless, in certain circumstances, acute hypoglycemia may increase individual vulnerability to the greater reward value of energy-dense foods.

This study is unique in that the effect of hypoglycemia on food preferences was evaluated in children and adolescents who did not have diabetes nor prior known experience of hypoglycemic episodes. Given that hypoglycemia is a recurrent problem for people living with insulin-dependent diabetes,^34^ standard practice entails education in carbohydrate intake control and treatment of hypoglycemic episodes. Therefore, the increase in desire for carbohydrate-rich foods reported by adults with type 1 diabetes when experiencing unblinded insulin-induced hypoglycemia^9^ may well reflect the application of explicit knowledge rather than the triggering of an automatic (instinctive) response.

Another strength of this study is that the computerised task enabled the evaluation of preference for high-carbohydrate foods in the context of other alternatives available, replicating the decision-making patients often face in everyday life. Adequate treatment of hypoglycemia requires the individual to process incoming information (ie, hypoglycemia symptoms), embed knowledge acquired during diabetes education, make value judgments on available food, and direct behaviour accordingly. If acute glucoprivation in the brain results in impaired cognitive function^6^ and counter-regulatory responses do not automatically guide the individual towards foods that facilitate rapid glucose absorption, it is reasonable that patients become vulnerable to opting for foods that they find particularly ‘tasty’ and overeating these foods.^4^ Our findings reinforce the importance for people with insulin-requiring diabetes and their carers to ensure that they have readily available ‘fast’ carbohydrates rather than rely on their instincts during hypoglycemic episodes.

In the context of pediatric diabetes care, it is also noteworthy that participants often selected the maximum number of gummy bears when asked to indicate how many they would like to eat (maximum=39), and this remained unaffected by glycemic state. These findings align with the experiences of some patients with diabetes who experience an uncontrolled nature of eating more than anticipated despite having preplanned a set quantity of food in case of a hypoglycemic episode. Our research has previously shown that primary school-aged children are able to predict future food intake using this computerised task.^20^ As gummy bears are experienced as appetizing foods by children, the present findings question whether they may be the most suitable treatment option. As participants in this study had not received diabetes education nor have had to treat a hypoglycemic episode before, it would be worthwhile testing the impact of prior knowledge on gummy bear selection by children and adolescents with type 1 diabetes experiencing a hypoglycemic episode. The choice of using gummy bears over other solutions (eg, dextrose tables) may be advantageous because children are more likely to eat them, but carers may have to ensure that access and quantity are limited to what is necessary for hypoglycemia treatment.

At diagnosis, children with type 1 diabetes and parents/guardians are told to carry a ‘hypo pack’ containing either dextrose tablets or prepacked gummy bears and a small carton of fruit juice, with the addition of food such as digestive biscuits to keep glucose levels in the recommended range after initial therapy. Individuals with long-term experience of type 1 diabetes and hypoglycemic episodes recognise that the recommended dose of dextrose/gummy bears can normalise glucose levels, but they often find it difficult not to overeat when treating a hypoglycemic episode and are having to inject additional insulin to correct afterwards. For some, this is triggered by anxiety around recurrent hypoglycemic episodes and intraindividual variation on how quickly episodes are detected. Findings from this study therefore provide confidence for people living with insulin-dependent diabetes that it is perfectly normal to find self-treating hypoglycemic episodes challenging, and reinforce the importance of ensuring that the ‘hypo pack’ is immediately available to ensure that food preference or cognitive impairment does not interfere with adequate treatment. As children become more independent in managing diabetes, education should also address fears associated with hypoglycemia, for example, by building confidence that following recommended treatment and monitoring blood sugar levels after treatment can prevent recurrent episodes.

In our attempt to discern carbohydrate-specific choice behavior, we were limited in the number of foods that were presented via the computerised task and we were unable to evaluate how other factors associated with food choice (eg, energy density) may have been influenced by hypoglycemia. As the research protocol was embedded within an existing clinical protocol, we were not always able to administer the computerised task when participants reached nadir venous glucose. This means that we were unable to control for the depth and duration of each participant’s hypoglycemia, which varies between individuals undertaking an ITT.^35^ However, it would have been unethical to subject children to hypoglycemia without a clinical indication; in this case, as part of an assessment of the pituitary adrenal axis. As the clinical procedure was prioritized, 10 recruited participants were withdrawn from the study. This means that our study was underpowered, and findings should be treated with caution. By running Bayesian data analysis on the null effects, we sought to examine the strength of the evidence as recommended elsewhere.^36^ We obtained moderate evidence of a null effect, and therefore we did not consider it appropriate to continue recruiting patients into the study.

In conclusion, the current study adds to accruing evidence that the physiological response to glucose depletion may be limited to triggering an urge to rapidly consume calories, with little capacity to direct food choice towards those foods that have a fast glucose absorption rate. Ensuring that the correct treatment is readily available and limiting access to alternative food options may be essential to avoid overeating in response to hypoglycemia.

## Data Availability

Data are available upon reasonable request.
