# Supplementary material for: Preference for high-carbohydrate foods does not change for children and adolescents in insulin-induced hypoglycemia
Source: BMJ Open Diabetes Res Care. 2022 Nov 8;10(6):e003065. doi: 10.1136/bmjdrc-2022-003065 (PMC9644309; doi:10.1136/bmjdrc-2022-003065)
Supplement: Supplementary data [file bmjdrc-2022-003065supp001.pdf]

Sauchelli, Rogers, Hamilton-Shield

Supplementary Material 1

Characteristics of the foods presented in the food preference task. The nutritional information has been derived from the packaging.

a) Non-vegetarian

Food	Energy density (kcal/g)	Kcal/g Fat	Kcal/g Carbs	Kcal/g Protein	Grams served (g)	Portion (kcal)
Rice with corn (60%/40%)	1.17	10.98	90.56	16.4	122.82	119.98
Banana	1.03	4.5	92.8	4.8	115.70	119.17
Chicken	1.06	9.9	0	96	113.00	119.78
Tomato & basil pasta	1.46	36.9	90.8	14.8	96.10	140.31
Strawberry trifle dessert	1.47	54	84	8	95.20	139.94
Boiled eggs	1.43	86.4	2	56.4	94.70	135.42
Noodles with katsu sauce (75%/25%)	1.86	55.8	116	10.8	91.00	169.26
Toffee and vanilla ice cream	1.84	58.5	113.2	11.2	88.30	162.47
Tuna and mayonnaise (85%/15%)	1.86	102.6	2	80.4	152.60	166.46
French fries	2.47	86.4	144.4	9.6	77.80	192.17
Apple pie	2.7	94.5	160	10.8	72.40	195.48
Beef burger	2.51	150.3	10.8	59.6	79.00	198.29
Naan bread	2.98	63	192.4	36	77.70	231.55
White bread with Nutella (75%/25%)	3.15	85.95	194.4	31	86.20	240.07
Dutch edam cheese	3.12	216	2	96	76.90	239.93
Ritz crackers	5	207	254	30.8	52.20	261.00
Twix	4.95	215.1	258.4	26.8	52.80	261.36
Pepperami sausage	5.01	396	8.8	104	51.40	257.51

b) Vegetarian

Food	Energy density (kcal/g)	Kcal/g Fat	Kcal/g Carbohydrate	Kcal/g Protein	Grams served (g)	Portion (kcal)
Rice with corn (60%/40%)	1.17	10.98	90.56	16.56	122.82	119.98
Banana	1.03	4.5	92.8	4.8	115.70	119.17
Quorn chicken	1.19	23.4	18	65.2	99.30	118.17
Tomato & basil pasta	1.46	36.9	90.8	14.8	96.10	140.31
Strawberry trifle dessert	1.47	54	84	8	95.20	139.94

Boiled egg	1.43	86.4	2	56.4	94.70	135.42
Noodles with katsu sauce (75%/25%)	1.86	55.8	116	10.8	91.00	169.26
Toffee and vanilla ice cream	1.84	58.5	113.2	11.2	88.30	162.47
Tuna and mayonnaise (85%/15%)	1.86	102.6	2	80.4	152.60	166.46
French fries	2.47	86.4	144.4	9.6	77.80	192.17
Apple pie	2.7	94.5	160	10.8	72.40	195.48
Quorn bacon style slices	2.14	141.3	21.6	40.4	88.20	188.75
Garlic bread	3.39	117	179.2	38.8	71.30	241.71
White bread with Nutella	3.46	112.41	199.2	29.6	78.60	235.75
Dutch Edam Cheese	3.12	216	2	96	76.90	239.93
Ritz crackers	5	207	254	30.8	52.20	261.00
Chocolate Digestives	4.95	212.4	248.8	26.8	52.10	257.90
Peanuts and Edamame beans (65%/35%)	4.7	336.6	28.8	86.8	104.30	262.25
