# Supplementary material for: Preference for high-carbohydrate foods does not change for children and adolescents in insulin-induced hypoglycemia
Source: BMJ Open Diabetes Res Care. 2022 Nov 8;10(6):e003065. doi: 10.1136/bmjdrc-2022-003065 (PMC9644309; doi:10.1136/bmjdrc-2022-003065)
Supplement: Supplementary data [file bmjdrc-2022-003065supp002.pdf]

Sauchelli, Rogers, Hamilton-Shield

Supplementary Material 2

Arterial glucose response to administration of insulin, split by (a) growth hormone (GH) and (b) cortisol sufficiency or deficiency based on insulin-tolerance test response.

(a)

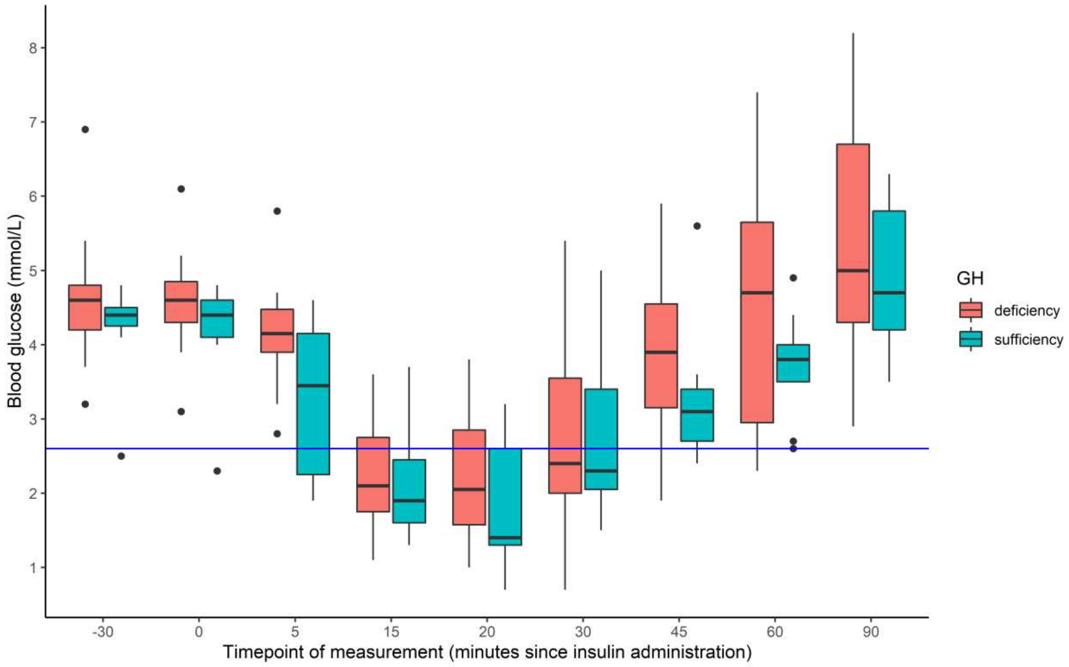

(b)

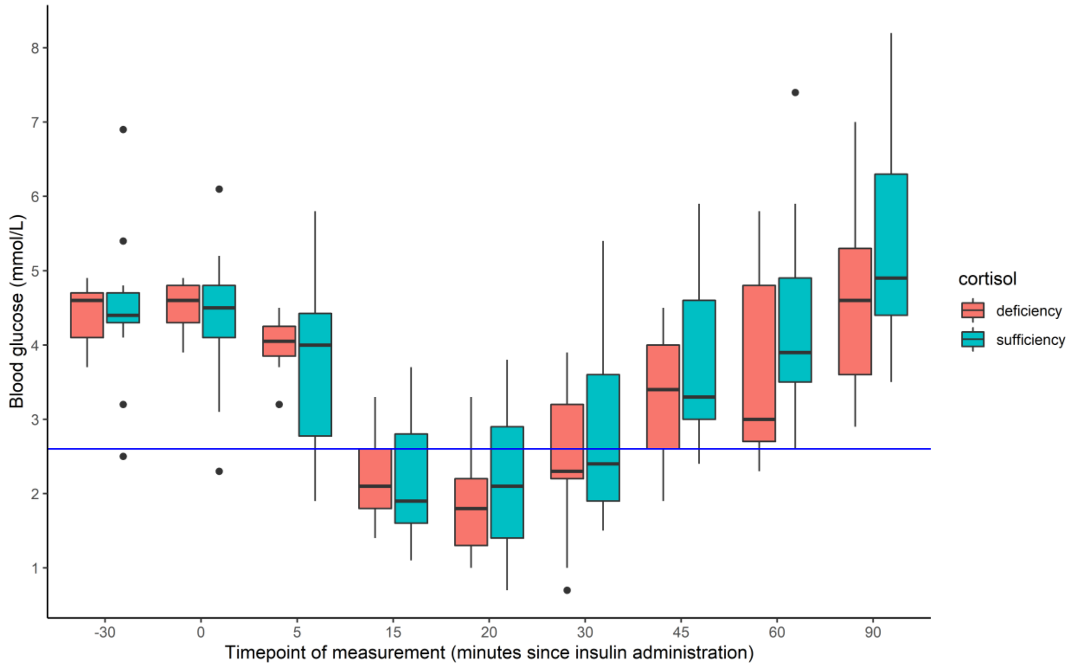
Blood glucose (mmol/L)
8
7
6
5
4
3
2
1
-30
0
5
15
20
30
45
60
90
Timepoint of measurement (minutes since insulin administration)
cortisol
deficiency
sufficiency
