# Supplementary material for: Preference for high-carbohydrate foods does not change for children and adolescents in insulin-induced hypoglycemia
Source: BMJ Open Diabetes Res Care. 2022 Nov 8;10(6):e003065. doi: 10.1136/bmjdrc-2022-003065 (PMC9644309; doi:10.1136/bmjdrc-2022-003065)

Sauchelli, Rogers, Hamilton-Shield

Supplementary Material 3

Food selection as a function of energy density

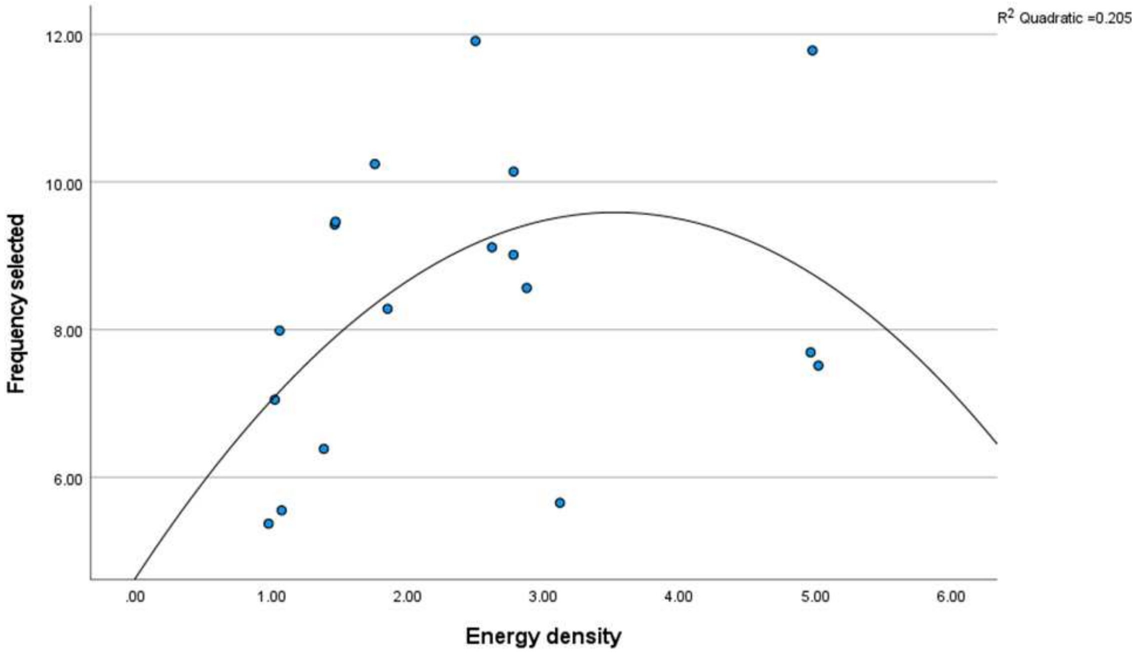

Supplement: Supplementary data [file bmjdrc-2022-003065supp003.pdf]
